# Transcranial Doppler Ultrasound, a Review for the Pediatric Intensivist

**DOI:** 10.3390/children9050727

**Published:** 2022-05-16

**Authors:** Marlina Elizabeth Lovett, Nicole F. O’Brien

**Affiliations:** Division of Critical Care Medicine, Department of Pediatrics, Nationwide Children’s Hospital, The Ohio State University College of Medicine, Columbus, OH 43205, USA; nicole.obrien@nationwidechildrens.org

**Keywords:** transcranial Doppler ultrasound, pediatric intensive care, neurocritical care

## Abstract

The use of transcranial Doppler ultrasound (TCD) is increasing in frequency in the pediatric intensive care unit. This review highlights some of the pertinent TCD applications for the pediatric intensivist, including evaluation of cerebral hemodynamics, autoregulation, non-invasive cerebral perfusion pressure/intracranial pressure estimation, vasospasm screening, and cerebral emboli detection.

## 1. Introduction

Pediatric neurocritical care is a rapidly evolving field as brain injury is associated with substantial morbidity and mortality [1,2]. The etiology of acute brain injury is diverse and may include varying disease pathologies due to injury to the brain itself (such as traumatic brain injury or stroke) or other organ dysfunction that has put the brain at risk [2]. Preventing secondary brain injury is a tenet of neurocritical care as it may worsen primary brain injury and may contribute to poor neurologic outcome [3]. As such, frequent assessment of the child’s neurologic status is critical and often incorporates both invasive and non-invasive neuromonitoring. Invasive neuromonitoring, such as intracranial pressure monitors, has associated risks of malposition, hemorrhage, and infection, and may not be available in all settings due to limited resources of equipment or personnel. Traditional modes of non-invasive neuromonitoring have focused on the neurologic exam, electroencephalogram data, and neuroimaging. Recently, technologies such as transcranial Doppler ultrasound (TCD) have become more commonplace and have provided a means to help elucidate cerebral hemodynamics in real-time at the bedside. This review will focus on the pertinent TCD applications for those who take care of critically ill children in the pediatric intensive care unit (PICU).

## 2. Brief Overview of Transcranial Doppler Ultrasound

Transcranial Doppler ultrasound was first reported by Rune Aaslid and colleagues in 1982. By using low-frequency ultrasound, applied through thin areas of bone, termed acoustic windows, it was possible to measure cerebral blood flow velocity (CBFV) in the major cerebral arteries [4]. By using TCD, it is possible to gain information regarding the patient’s cerebral hemodynamics in real time at the bedside. This non-invasive imaging may assist with the identification of acute intracranial processes and may allow for trends pre- and post-intervention. As such, TCD can augment the patient’s clinical exam and may be impactful for the treating intensivist.

**Figure 1:**
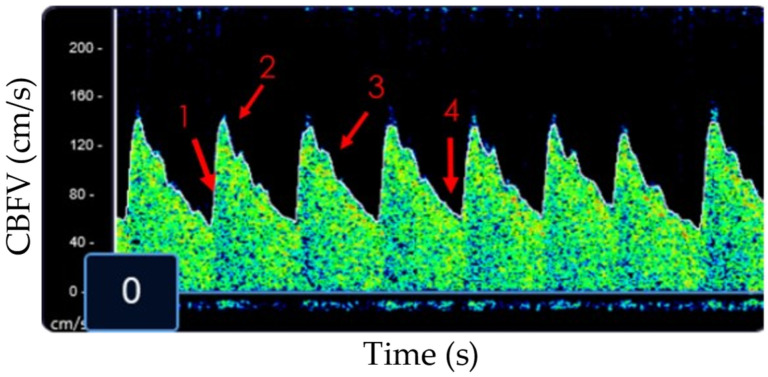
Example of a normal MCA waveform on a non-imaging TCD machine. 1: Beginning of systole; 2: peak systole; 3: dichrotic notch; 4: end-diastole. CBFV: cerebral blood flow velocity.

The four acoustic windows allow the sonographer to investigate cerebral hemodynamics in both the anterior and posterior circulation. The transtemporal window (the primary window of the anterior circulation) allows for evaluation of the middle cerebral artery (MCA), the anterior cerebral artery (ACA), the posterior cerebral artery (PCA), and the intracranial portion of the internal carotid artery (ICA). The transorbital window allows for evaluation of the ophthalmic vessels and the carotid siphon. The submandibular window allows for evaluation of the extra-cranial portion of the internal carotid artery (Ex-ICA). Lastly, the suboccipital/transforaminal window is used to assess the vertebrobasilar system of the posterior circulation. A complete TCD exam will evaluate all of the vessels identified above but a more focused exam of a critically ill child will often be limited to the anterior circulation (MCA), the extra-cranial carotid (Ex-ICA), and potentially the vertebrobasilar system based upon the clinical question [5]. Data reported by the TCD machine include at a minimum CBFV (systolic [Vs], diastolic [Vd] and mean [Vm] flow velocities, Figure 1) and machine-derived variables that may be helpful to understand cerebrovascular resistance, such as the pulsatility index (PI, PI = [Vs − Vd]/Vm).

There are two general categories of TCD machines available, non-imaging (non-duplex) and imaging (duplex) devices. Imaging TCD combines Doppler data with a cross-sectional view of the area being insonated [6]. This allows for rapid identification of the major cerebral arteries. Vessel identification through the use of non-duplex, or non-imaging TCD is based upon the depth of sample volume, direction of blood flow identified, the spectral display, and the characteristic sound of each vessel. While the majority of the reported TCD literature in critically ill children focuses on non-imaging TCD, the use of imaging TCD is becoming more frequent as it is accessible on most commercially available ultrasound machines used in the PICU. In a recent neuromonitoring practices survey, of respondents who reported using TCD, 18 (86%) used imaging TCD whereas 5 (24%) used non-imaging TCD [7]. Given the focus of the current literature base, most studies reported in this manuscript use non-imaging TCD.

Interpretation of TCD data is dependent on a myriad of factors. Cerebral blood flow velocity is known to increase in childhood, peak near age 6 years, then slowly decline to adult levels. Therefore, in children, it is essential to compare measured values to published normative values for age [5]. Measured CBFV is considered abnormal if the value is greater than or less than 2 standard deviations from the previously published normative data [8]. Alterations in cerebral blood flow velocity compared to age-appropriate normative data have been associated with outcome in varying disease processes noted in critically ill children. Interpretation of TCD data is most impactful if the interpreter also evaluates the current physiologic state of the patient. Fever is known to increase cerebral metabolic demand and is ultimately associated with elevated CBFV. Other conditions also associated with hyperemia (elevated CBFV) include: anemia, hypertension, elevated partial pressure of carbon dioxide, and seizures. Conversely, shock and hypocapnia have been associated with reduced CBFV. Sedative medications, frequently used in the intensive care unit, have been associated with reduced CBFV in the absence of intracranial pathology [9].

## 3. Applications in the Pediatric Intensive Care Unit

The use of TCD in the PICU has been gaining popularity with a recent study reporting that 93% of surveyed institutions (*n* = 27/29) providing pediatric neurocritical care use TCD [10]. In contrast, another survey of 52 institutions noted that 40% (*n* = 21/52) now use TCD (responding institutions likely overlapped) [7]. The increasing popularity of TCD has prompted additional work to provide recommendations to standardize the approach to performing, interpreting, and reporting TCD [5].

The literature base for the use of TCD in the PICU is limited but quickly growing. Its use has been described in a variety of clinical scenarios including traumatic brain injury, hypoxic-ischemic brain injury, stroke, sepsis, diabetic ketoacidosis, central nervous system infections, hepatic encephalopathy, and monitoring while on extra-corporeal membrane oxygenation. Use is institution-dependent but is more frequently driven by physician recommendation as opposed to institutional protocols [7]. A recent multi-center study of pediatric intensivists in France noted that TCD was used for diagnosis, to guide management, and as a prompt to obtain additional imaging [11]. TCD has also been used in the pre-hospital setting in adult patients to identify large vessel occlusions and aid in the management of severe TBI patients, although this has not yet been applied in the pediatric setting and warrants additional investigation [12,13].

Given the heterogeneity of disease-specific TCD use, it is likely more impactful to consider the indication for TCD use. Expert consensus recommendations state, “any patient in the PICU with concern for pathophysiological changes to cerebral hemodynamics is a candidate to undergo TCD examination” [5]. The current literature base for TCD in the PICU, can be broken down into a few main categories of evidence: (1) evaluating cerebral hemodynamics, (2) investigating cerebral autoregulation and cerebral vasoreactivity, (3) evaluating intracranial pressure and cerebral perfusion pressure, (4) screening for cerebral vasospasm, and (5) emboli monitoring.

### 3.1. Evaluating Cerebral Hemodynamics

Small studies in children with traumatic brain injury (TBI) have begun to investigate the association of altered CBFV with outcome. In 36 children with moderate to severe TBI, an end-diastolic flow velocity (Vd) < 25 cm/s on arrival to the emergency department was associated with poor outcome [14]. Similarly, in 69 children with moderate to severe TBI an abnormally low MCA CBFV (<2 standard deviations from normative) was identified in 6% of children, none of whom had a good neurologic outcome [15]. These two small studies together suggest that abnormally low CBFV may be associated with poor neurologic outcome following trauma. It is unclear if interventions should be taken to normalize TCD data. However, TCD data may be helpful at the bedside when determining the safety of specific intracranial hypertension treatment recommendations. For example, if a child has abnormally low CBFV, it may give pause to perform hyperventilation as that would cause further reduction in CBFV.

Cerebral hemodynamics have also been investigated in children with hypoxic-ischemic brain injury using TCD. In a small study of 26 children with hypoxic–ischemic brain injury, abnormal CBFV within 48 h of injury was associated with poor neurologic outcome [16]. No patient with a favorable neurologic outcome had abnormal CBFV on day 1, yet 38% of those in the unfavorable outcome group had abnormal CBFV (*p* = 0.03). A recent pilot study of 21 pediatric survivors of asphyxial out-of-hospital cardiac arrest who underwent therapeutic hypothermia used point-of-care TCD to guide therapy [17]. TCDs were categorized into three groups: (1) continuous waveform with a PI < 1.1 and a normal or high Vm, (2) continuous waveform with PI between 1.1–2 with low or normal Vm, and (3) discontinuous waveform or continuous waveform with PI > 2.0 and low Vm. Osmolar therapy was used with varying levels based upon TCD category and the goal of TCD guided care was “to achieve a continuous waveform and at least the lower limit of Vm, to maintain the serum osmolality at 320–340 mOsm/kg H_2_O and serum sodium 155–160 mEq/L.” The one-month survival rate of those who underwent TCD-guided treatment (*n* = 12) was higher than those who did not (*n* = 9) undergo point-of-care TCD guided therapy (75% vs. 22.2%, *p* = 0.03). Additional work with larger sample sizes is needed to truly understand how TCD can be incorporated into the care of children post-cardiac arrest.

TCD has been used to evaluate cerebral hemodynamics for children on extra-corporeal membrane oxygenation (ECMO). Children on ECMO were noted to have lower CBFV compared to normative data [18,19,20]. In a small study of 18 children on ECMO, 4 developed acute cerebral hemorrhage and these children had abnormally high CBFV [18]. Vs was 123 ± 8% of the predicted value, Vd was 130 ± 18% of the predicted value, and Vm was 127 ± 9% of the predicted value. In these four children, TCD elevation preceded clinical detection of acute intracranial hemorrhage by 2–6 days. A multi-center study was performed to further investigate these findings. In 52 children supported on ECMO, there was a reduction in CBFV that was most prominent in children who were cannulated peripherally (*n* = 39) compared to centrally (*n* = 13) [19]. For those with peripheral cannulation, right MCA Vs was 1.1 ± 0.16 standard deviations (SD) below normal, left MCA Vs was 1.2 ± 0.16 SD below normal, whereas with central cannulation, right MCA Vs was 0.58 ± 0.08 SD, and left MCA Vs was 0.64 ± 0.08 SD below normal (*p* = 0.01). There was no difference in Vd. Eight children developed cerebral ischemia, six of which were under 3 months of age. These infants were noted to have a higher PI than children of similar age on ECMO without acute neurologic injury [19]. No child developed an acute intracranial hemorrhage within this cohort. TCD may be helpful to assist in the detection of acute neurologic injury for the child on ECMO but additional work is needed.

TCD has been used to investigate sepsis-associated encephalopathy in a small cohort of children [21]. The included 45 children had severe sepsis or septic shock and were encephalopathic without associated primary brain injury. TCD was performed on the day of admission after the initial resuscitation with a blood pressure goal of at least the 5th percentile for age. When analyzed by survival, non-survivors had markers of elevated cerebrovascular resistance (higher pulsatility index 1.33 [IQR 1.16–1.83] vs. 0.99 [0.8–1.1], *p* < 0.001; and higher resistive index 0.75 [0.67–0.82] vs. 0.61 [0.69–0.67], *p* < 0.001). The authors found that as the patient’s clinical exam deteriorated (worsening Full Outline of Unresponsiveness [FOUR] score), this was inversely correlated with the PI. Additional work is needed to evaluate the use of TCD in both the acute resuscitation of the child with sepsis associated encephalopathy as well as its potential association with outcome.

**Figure 2:**
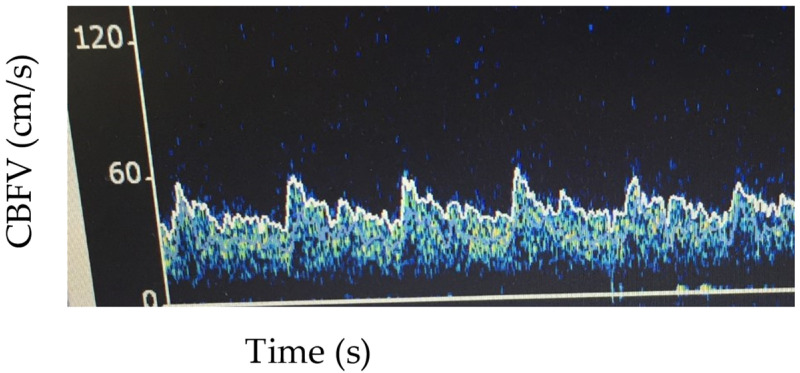
Example of MCA low flow pattern. Vs: 55 cm/s, Vm 42 cm/s, Vd 33 cm/s, pulsatility index 0.54.

As opposed to solely reporting CBFV, there has been some use of reporting patterns seen on TCD. In a study of 47 African children with bacterial meningitis without sickle cell anemia or cerebral malaria, children were divided into categories based on TCD CBFV and the PI [22]. In the normal-flow category, TCD values were within 2 standard deviations of age-based normative values whereas in the high flow group Vs, Vm were > 2 SD above normative value and further categorized based upon PI being abnormally low (PI < 0.6), normal (PI 0.6–1.3), or abnormally high (PI > 1.3). In the low-flow group, Vs and Vm were < 2SD below normal and were further characterized based upon a normal or abnormal PI. These patterns were then grouped as either low-risk (normal flow or high flow/normal PI) or high-risk (high flow/low PI or low-flow [Figure 2 for example of low flow]). Twenty-one percent of children had a normal TCD on admission and all had a good neurologic outcome on hospital discharge. Children with a high-risk pattern more frequently had poor neurologic outcome (82 vs. 38%, *p* = 0.001).

Similarly, in a study of children with cerebral malaria, TCD patterns were identified as hyperemia (Vm ≥ 2 SD from normative, Lindegaard Ratio < 3, and absent dicrotic notch on waveform analysis), low flow (Vd/Vs/Vm all < 2 SD below normative and PI < 1.2), microvascular obstruction (Vs normal, Vd < 2 SD from normative, and PI ≥ 1.2), cerebral vasospasm (Vm ≥ 2 SD from normative, Lindegaard Ratio ≥ 3, and dicrotic notch present on waveform analysis), and isolated posterior hyperemia (Vm in the basilar artery ≥ 2 SD from normative and Vm in the MCA was normal) [23]. Of the 160 children with cerebral malaria, 35 (22%) had microvascular obstruction, 42 (26%) had hyperemia, 21 (13%) had vasospasm, 46 (28%) had low flow, 7 (4%) had isolated posterior hyperemia, and 5 (3%) had normal flow. Children with low flow were most at risk to die [23]. These identified patterns may provide guidance to the clinician for potential therapeutic options.

### 3.2. Investigating Cerebrovascular Autoregulation

Cerebral autoregulation is a complex physiologic process that involves changes in cerebrovascular resistance (vasodilation or vasoconstriction) in response to pressure changes to maintain constant cerebral blood flow. This process may be impaired or absent in a number of neurocritical care conditions [24]. Autoregulation can be investigated using both intermittent and continuous TCD data. One method of investigating autoregulation using intermittent data is by calculating the transient hyperemic response ratio (THRR, THRR = FV_hyperemia_/FV_baseline_) after dynamically challenging the cerebral vasculature. Intact autoregulation in the adult literature is defined as THRR ≥ 1.1 [25,26]. However, in a small cohort of critically ill, intubated, sedated children without acute neurologic injury, the median THRR was 1.035 [16]. When using continuous TCD and an arterial line, one can evaluate the relationship between the mean arterial blood pressure and Vm, termed Mx. When Mx is 0 or negative, this would suggest that autoregulation is intact. In theory, the most negative Mx would occur at the optimal mean arterial blood pressure. This is an area ripe for investigation and could theoretically help guide post-resuscitation care of the child with acute traumatic or atraumatic brain injury.

The THRR has been investigated in a small cohort of 26 children with HIE [16]. No child with a favorable neurologic outcome consistently had a THRR ≥ 1.1. Twenty-three percent of children with a favorable neurologic outcome had a THRR ≥ 1.1 on their final study, whereas no child with an unfavorable outcome had a THRR ≥ 1.1 on their final study. Sixty-three percent of TCD studies in children with a favorable neurologic outcome had a THRR ≥ 1.035 whereas 25% of studies in the unfavorable neurologic outcome had a THRR ≥ 1.035 (*p* < 0.0001) [16].

In children with cerebral malaria, those with a normal neurologic outcome had a higher THRR compared to those with poor neurologic outcome (1.09 vs. 0.98, *p* ≤ 0.001) [23]. This finding was also present in children with bacterial meningitis (THRR of 1.12 ± 0.06 for children with a good neurologic outcome vs. 0.98 ± 0.05 for those with a poor neurologic outcome, *p* = 0.03) [22].

### 3.3. Investigating Cerebral Vasoreactivity

In contrast to cerebral autoregulation, cerebral vasoreactivity represents the ability of the cerebral vasculature to dilate or constrict in response to specific vasoactive stimuli, such as carbon dioxide [24]. Hypercapnea results in vasodilation of the cerebral arterioles whereas hypocapnea results in arteriolar vasoconstriction [6]. This phenomenon can be measured using TCD by controlling ventilation either with the use of the breath-holding index (BHI) or adjusting the ventilator. The BHI involves measuring CBFV with normal inspiration, asking the patient to hold their breath for 30 s and recording CBFV again during the 10 s period following breath holding. While this may be insightful for non-critically ill children, often critically ill children are on invasive or non-invasive ventilation that may make this maneuverable challenging. For children receiving invasive mechanical ventilation, it is possible to manipulate the minute ventilation, often through manipulation of the respiratory rate to measure carbon dioxide reactivity (CO_2_R). This can be performed by measuring CBFV at baseline, manipulation of the ventilator to lower the end-tidal CO_2_ by at least 5 mmHg, maintaining stability for at least 2 min, then re-measuring CBFV. CO_2_R is calculated by: %∆Vm/mmHg [27]. However, this must be carried out in a thoughtful manner to avoid potential harm in children as sustained hypocapnia may be associated with cerebral ischemia, which may be harmful in children with acute brain injury.

Carbon dioxide reactivity (CO_2_R) has been investigated in a small cohort of children with moderate and severe TBI (*n* = 38) [27]. Children were divided into three age groups: <2 years, 2–5 years, and >5 years. CO_2_R was highest in the 2–5-year age group. A CO_2_R < 2.7% was considered abnormal and most predictive of poor outcome. Most children had an abnormal CO_2_R for greater than 20% of measurements, most evident in those with severe TBI who had abnormal values for 35% of the time versus those with moderate TBI who had abnormal values for 10% of the time. It was also noted that CO_2_R improved over time with normalization in most patients by post-injury day 3 or 4. Additional research is needed to further investigate associations of abnormal CO_2_R and outcome in a thoughtful manner.

### 3.4. Evaluating Intracranial Pressure and Cerebral Perfusion Pressure

TCD has been investigated as a potential means to evaluate for elevated intracranial pressure (ICP). Invasive ICP monitors have known risks including: intracranial hemorrhage, malposition, and infection. Given these risks, there has been interest in using TCD as a non-invasive method in which to evaluate for elevated ICP or to potentially risk-stratify patients that may benefit from invasive ICP monitor use.

**Figure 3:**
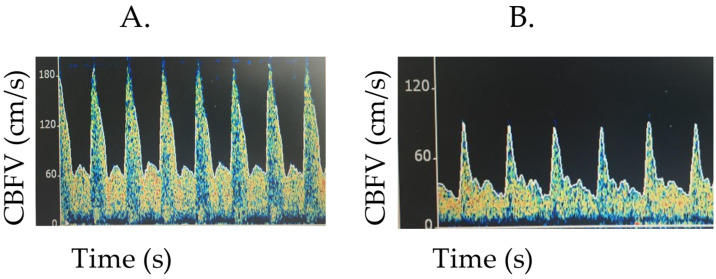
Examples of TCDs with concern for elevated intracranial pressure. (**A**). The child’s Vs is maintained with a reduction in Vd, leading to a subsequent increase in the PI. Flow velocities (cm/s), Vs 167, Vm 76.6, Vd 56.2, PI 1.43. (**B**). In this image, Vd is more reduced than Vs, also with an increase in PI. Flow velocities (cm/s): Vs 90.1, Vm 43.1, Vd 26.9, PI 1.45.

As the ICP rises, there is a more pronounced reduction in Vd than the systolic flow velocity (Vs). This leads to a rise in the pulsatility index (PI, PI = (Vs − Vd)/Vm). The pulsatility index has been investigated at varying time points in children with severe TBI. The literature, while limited by small sample sizes, suggests that the pulsatility index may be most impactful in the early stage of illness. TCDs performed on the day of admission in 117 children were characterized as being abnormal if they had evidence of flow reversal, no flow, reduction in Vd (Vd < 25 cm/s), or an elevated pulsatility index (PI > 1.31). An abnormal TCD had 94% sensitivity for predicting an ICP > 20 mmHg [28]. Similarly, in 36 children with severe TBI, PI ≥ 1.3 on post-injury day 0 or 1 had 100% sensitivity and 82% specificity for ICP ≥ 20 mmHg [29]. Beyond day 1 of injury, the sensitivity of PI ≥ 1.3 to detect intracranial hypertension fell to 47%. Therefore, limited data suggest the TCD-derived PI may be helpful in the detection of intracranial hypertension early in the course of severe TBI. Conversely, the PI was recently investigated within a heterogenous cohort of 30 children with ICP monitors placed (67% infectious, 10% metabolic, 17% immune-mediated, and 7% other) [30]. Although children with intracranial hypertension had an elevated PI (0.92 ± 0.41) compared to those measured points without intracranial hypertension (0.69 ± 0.27, *p* < 0.001), the suggested cut-off for a PI value reflecting ICP ≥ 20 mmHg was 0.51 with 89% sensitivity, 10% specificity, 50% positive predictive value, and 47% negative predictive value. The AUC was 0.571 (95% CI 0.492–0.649). It should be noted that the PIs reported were lower than previously published normative values for intubated and sedated critically ill children [9]. It is unclear if the PI is more impactful in specific disease processes, but at this time the evidence does not show a reliable association between PI and intracranial hypertension in children (see Figure 3 for example of elevated PI).

In addition to using Vd and the pulsatility index, there has been interest in trying to evaluate for intracranial hypertension and calculate the cerebral perfusion pressure (CPP) non-invasively. Using continuous TCD in adults with head injury, Czosnyka et al. was able to calculate an estimated CPP (CPPe = [MAP × (Vd/Vm)] + 14). In their work, the correlation between CPP and CPPe was 0.73, with an average absolute error of 6.5 mmHg [31]. This formula was applied to a small study of 23 children with severe TBI using intermittent TCD data and was not successful. The average discrepancy was 3.7 mmHg but limits of agreement were −17 to +25 mmHg [32]. A similar study was performed applying the same formula to a group of 10 children with acute brain injury requiring an ICP monitor (*n* = 3 with TBI, *n* = 7 with intracranial hemorrhage) to non-invasively estimate ICP based on intermittent TCD data [33]. In this study, a correlation was found between the invasive ICP and the non-invasive estimated ICP (r = 0.441, *p* < 0.0001). However, bias was 7.91 mmHg with 95% CI of ±16.26 mmHg. Taken together, these studies suggest that using the CPPe formula and subsequent estimated ICP, based on intermittent TCD data in children is likely inaccurate. There have been other complex mathematical formulas and modeling that have been able to use continuous TCD to estimate CPP successfully, but they are likely out of the scope of the pediatric intensivist at the bedside [34,35].

### 3.5. Vasospasm Screening

In adults, detection and management of cerebral vasospasm following aneurysmal subarachnoid hemorrhage (SAH) is an essential component of care to prevent delayed cerebral ischemia and reduce subsequent morbidity and mortality. In adult aneurysmal SAH management, TCD has become a bedside non-invasive screening tool as it may allow for the detection of cerebral vasospasm before it becomes clinically apparent [36]. The American Heart Association gives a class IIa recommendation to the statement that, “Transcranial Doppler (TCD) is reasonable to monitor for the development of arterial vasospasm” [37]. A recent meta-analysis in adults showed that TCD had a 93.7% positive predictive value for cerebral vasospasm [38]. Adult TCD-based criteria to define vasospasm have been well defined and include thresholds for mean flow velocity (Vm) and the Lindegaard ratio (LR). The LR is the ratio of the Vm in the MCA to the extracranial portion of the ipsilateral internal carotid artery. Mild vasospasm is defined as a MCA MFV of 120–149 cm/s and a LR of 3–6, moderate vasospasm is a MFV 150–199 cm/s and a LR of 3–6, and severe vasospasm is defined as MFV > 200 cm/s and LR > 6 [39]. Given that healthy children have higher baseline CBFV than adults, there is concern that application of the adult criteria to children would overestimate the true incidence of cerebral vasospasm.

Due in part to this lack of standard definitions, the incidence of cerebral vasospasm in children with acute neurologic illness or injury is largely unknown and may be an under-recognized entity. There are a number of different TCD-based criteria used in the pediatric literature to suggest vasospasm. These range from adult-based criteria to variable criteria including Vm and some with or without the Lindegaard ratio. The most frequently reported criteria include: MCA Vm ≥ 2 standard deviations above age-based normative values and a LR ≥ 3 [15,40,41]. However, these studies did not have angiographic correlations. Current pediatric expert recommendations state, “No Lindegaard Ratio has been validated in children to differentiate between hyperemia and vasospasm in the MCA and thus using specific cut-offs for diagnosing, grading, or determining the clinical significance of vasospasm in the MCAs cannot be recommended. However, following LR values over time may have clinical utility to determine trends in cerebral blood flow” [5].

**Figure 4:**
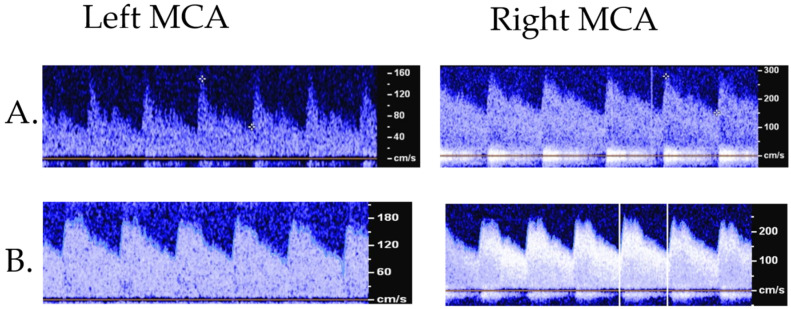
Example of TCD with concern for vasospasm (**A**) versus hyperemia (**B**). A: 11-year-old male with an arteriovenous malformation that developed concern for vasospasm on TCD which was confirmed on additional imaging. Flow velocities (cm/s): right MCA—Vs 280, Vm 195 (+9.7 SD from normative), Vd 152, LR: 3.7; left MCA—Vs 150, Vm 90 (+0.92 SD), Vd 59.7, LR: 1.6. B: 11 yo female with an MCA aneurysm, TCD concerning for hyperemia. Flow velocities (cm/s): right MCA—Vs 254, Vm 170 (+7.5 SD), Vd 129, LR: 1.45; left MCA—Vs 220, Vm 142 (+5.25 SD), Vd 104, LR: 1.49. Vs: systolic flow velocity, Vm: mean flow velocity, Vd: diastolic flow velocity; LR: Lindegaard ratio.

To begin to use TCD as a non-invasive tool to detect the presence or absence of cerebral vasospasm in children, we need to not only establish TCD-based criteria for pediatric vasospasm that would demonstrate sufficient concordance with angiographic and other neurovascular-imaging modalities, but we must also understand how cerebral vasospasm affects the clinical outcome in children following acute brain injury. While TCD screening after SAH has not reached a guideline recommendation for consideration in pediatric neurocritical care practice, several studies indicate that children with SAH may be at high risk for developing vasospasm. In a report of 43 children with ruptured cerebral aneurysms, 19 underwent angiogram between days 3–17 with a 53% incidence of vasospasm with no mortality difference in the entire cohort between those with vasospasm and those without [42]. In 37 children with SAH who underwent diagnostic angiograms, 17 (45%) showed evidence of vasospasm [43]. Many pediatric patients with vasospasm may not develop symptoms of delayed cerebral ischemia (DCI). Of the 17 children with angiographic evidence of vasospasm, 14 were asymptomatic. Although long-term outcomes were not evaluated based upon the presence or absence of vasospasm, all three children with symptomatic vasospasm had a good long-term outcome [43]. This finding should not be interpreted that cerebral vasospasm does not contribute to the development of DCI in children. Garg et al. reported that following aneurysmal SAH, 4 out of 10 children developed new cerebral infarcts attributable to vasospasm [44]. The clinical significance of vasospasm remains unclear as there are limited data on cerebral vasospasm rates and delayed cerebral ischemia in children (see Figure 4).

### 3.6. Emboli Monitoring

**Figure 5:**
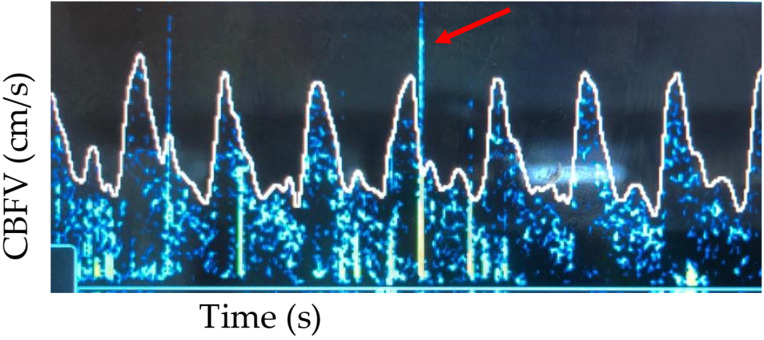
Multiple examples of HITS detected (red arrow for a singular example).

The use of continuous TCD allows for a non-invasive way to detect cerebral emboli in real time. Microemboli (thrombus, air, lipid, platelet aggregates, etc.) produce high-intensity transient (HITS) signals on TCD [45]. These are transient, lasting less than 300 msec, high amplitude (3 decibels higher than the background flow signal), unidirectional on the Doppler spectrum, and have an audible signal (snap, chirp) [46]. These signals have been detected in cardiac catheterization, cardiac surgery, and during ECMO [45,47,48]. To detect HITS, the TCD probe must remain in a stable position, often requiring the use of headgear to secure the probe. The detection of HITS may be automatic with commercially available software, or manual, which is often laborious and time-consuming [49]. Additional work is needed to improve automated detection of micro-emboli. At this point, the clinical significance of micro-emboli detected on TCD is unclear. It is not yet known if micro-emboli have a clear association with additional neurologic injury or if their detection prompts intervention or additional imaging (see Figure 5 for an example of HITS).

## 4. Limitations of TCD

Given the increased use of TCD in the PICU, it is important for the pediatric intensivist to be aware of its limitations. The validity of data derived from TCD is operator dependent and the operator must have an understanding of cerebrovascular anatomy, physiology that impacts TCD findings, and the current physiology of the patient. The current literature base regarding the use of TCD in critically ill children is often limited by small sample sizes, heterogeneity in patient population, and varying interpretations of TCD data. Standardization of the TCD exam has been previously described and will be important for any additional work [5].

## 5. Future Directions

As the use of transcranial Doppler ultrasound is becoming more frequent in the pediatric intensive care unit and is more easily accessible with point-of-care ultrasound machines, we anticipate that the literature base will continue to grow in the coming years. We anticipate that there will be continued work in the applications highlighted in this manuscript, but we also suspect that there will be investigation into waveform analysis and work to integrate TCD with multi-modal neuromonitoring to allow the most informed decision making. Furthermore, with the bedside applications, it may be possible to incorporate TCD data into acute resuscitations, integrate the data for decisions regarding escalation or de-escalation in care, and prompts for additional neuroimaging. We have only scratched the surface on the use of TCD at the bedside in the PICU and we suspect that as more intensivists use point-of-care ultrasound, TCD will become even more widespread.

## Data Availability

Not applicable.
